# Synthesis and biological evaluation of chrysin derivatives containing α-lipoic acid for the treatment of inflammatory bowel disease

**DOI:** 10.3389/fchem.2024.1406051

**Published:** 2024-05-27

**Authors:** Pengyu Zhao, Yusen Hou, Tingting Yan, Jie Kang, Ye Tian, Jiaxin Li, Chenjuan Zeng, Funeng Geng, Qi Liao

**Affiliations:** ^1^ School of Clinical Medicine, Chengdu University of Traditional Chinese Medicine, Chengdu, China; ^2^ School of Pharmacy, Chengdu University of Traditional Chinese Medicine, Chengdu, China; ^3^ State Key Laboratory of Southwestern Chinese Medicine Resources, College of Pharmacy, Chengdu University of Traditional Chinese Medicine, Chengdu, China; ^4^ Sichuan Key Laboratory of Medical American Cockroach, Chengdu, China; ^5^ Yunnan Shengke Pharmaceutical Co., Ltd., Kunming, China; ^6^ Guizhou Yunfeng Pharmaceutical Co., Ltd., Xingyi, China; ^7^ Sichuan Engineering Research Center for Medicinal Animals, Chengdu, China

**Keywords:** inflammatory bowel disease, chrysin derivatives, small molecules, anti-inflammatory activity, TNF-α

## Abstract

This study introduces newly discovered chrysin derivatives that show potential as candidate molecules for treating inflammatory bowel disease (IBD). Compound **4b**, among the synthesized compounds, displayed significant inhibitory effects on monocyte adhesion to colon epithelium induced by TNF-α, with an IC_50_ value of 4.71 μM. Further mechanistic studies demonstrated that **4b** inhibits the production of reactive oxygen species (ROS) and downregulates the expression of ICAM-1 and MCP-1, key molecules involved in monocyte-epithelial adhesion, as well as the transcriptional activity of NF-κB. *In vivo* experiments have shown that compound **4b** exhibits a dose-dependent inhibition of 2, 4, 6-trinitrobenzenesulfonic acid (TNBS)-induced colitis in rats, thereby validating its effectiveness as a colitis inhibitor in animal models. These results indicate that **4b** shows considerable promise as a therapeutic agent for managing IBD.

## 1 Introduction

The pathogenesis of inflammatory bowel disease (IBD) is a multifaceted process that encompasses the interplay of genetic predisposition, environmental triggers, and immune dysregulation within the gastrointestinal system (Seyedian S et al., 2019). The etiology of the disease involves alterations in the innate immune response of the body. TNF-α functions to induce inflammation by stimulating the synthesis of additional pro-inflammatory cytokines and adhesion molecules, facilitating the attachment and transmigration of white blood cells across the intestinal epithelium (Jang D et al., 2021). The critical phase in the development of inflammation and tissue damage seen in IBD entails the influx of additional inflammatory cells into the compromised intestinal epithelium (Saez A et al., 2021; Wang J. et al., 2023). To date, TNF-α inhibitors have demonstrated significant efficacy in managing chronic inflammatory conditions, such as IBD, through their ability to suppress the expression of pro-inflammatory cytokines and adhesion molecules (Peng J et al., 2014; Willrich et al., 2015). Currently, the most effective therapeutic approach for individuals diagnosed with IBD involves the use of anti-tumor necrosis factor-α (anti-TNF-α) antibodies, such as infliximab. However, the known toxicities of the antibody likely hamper its clinical deployment, encompassing infections, immunosuppression, and malignancies (Hemperly and Vande Casteele, 2018; Parigi et al., 2021). Hence, the advancement of more potent and secure medications is imperative for the treatment of IBD.

Natural products are considered to be valuable resources for the initiation of drug discovery (Li et al., 2016; Atanasov et al., 2021). Chrysin, a naturally occurring flavonoid, exhibits a diverse range of biological activities including antibacterial, anti-tumor, antioxidant, anti-allergic, and anti-inflammatory properties (Naz et al., 2019; Oršolić et al., 2022). Extensive evidence supports the notion that chrysin exerts a wide array of anti-inflammatory effects by targeting multiple molecular pathways and their associated targets (Zeinali et al., 2017). Specifically, chrysin possesses anti-inflammatory effects by reducing the production of pro-inflammatory cytokines TNF, IL-1, and IL-6 (Ahad et al., 2014; Faheem et al., 2023). However, the therapeutic effectiveness of chrysin has been hindered by its inadequate aqueous solubility and low bioavailability (Li Y et al., 2019; Bhowmik et al., 2022). Consequently, numerous chrysin derivatives have been synthesized with the aim of augmenting their bioactivities under physiological circumstances (Wang Q et al., 2014; Ghorab et al., 2023). Especially, Chen *et al.* conducted the preparation of various chrysin derivatives incorporating aromatic substituents or long-chain aliphatic hydrocarbons (Chen et al., 2020). Likewise, Li *et al.* prepared a collection of chrysin derivatives featuring diverse amino acid species. In comparison to the parent molecule, these derivatives exhibited enhanced *in vitro* bioactivities (Li Y et al., 2021). These studies have illuminated the importance of chrysin derivatization as a promising method in the development of more effective treatment strategies.

α-Lipoic acid (α-LA) is a ubiquitous biological antioxidant that traverses the blood-brain barrier and serves as a cofactor for enzymes crucial to cellular metabolism (Salehi et al., 2019). Its remarkable ability to neutralize free radicals and uphold cellular oxidoreductive equilibrium has garnered significant attention, particularly in relation to its potential therapeutic efficacy in various ailments, including IBD (Wang Z et al., 2022). In particular, α-LA derivatives bearing the indoles scaffold demonstrate notable anti-inflammatory efficacy by inhibiting the production of pro-inflammatory factors such as nitric oxide (NO) and inducible nitric oxide synthase (iNOS) in lipopolysaccharide (LPS) and interferon gamma (IFNγ)-stimulated RAW 264.7 macrophages (Prieto-Hontoria et al., 2016). Additionally, the *in vivo* studies provided evidence that α-LA effectively attenuated the concentrations of TNF-α. Therefore, considering the functional specificity of α-LA, the incorporation of this compound into chrysin holds the potential to enhance both the bioactivities and physicochemical properties.

In this study, a series of chrysin derivatives were prepared through introducing α-LA functional group. Next, all synthesized compounds were subjected to initial *in vitro* screening to assess their potential anti-inflammatory effects on the adhesion of monocytes to colon epithelial cells induced by TNF-α. Among them, **4b** was identified as a promising candidate molecule. *In vitro* and *in vivo* experiments have shown that **4b** exhibits notable inhibitory effects on TNF-α-induced adhesion of monocytic-colonic epithelial cells. The aforementioned findings collectively indicate that **4b** possesses the potential to serve as a lead molecule in the therapeutic intervention of IBD.

## 2 Results and discussion

### 2.1 Design and synthesis

**SCHEME 1:**
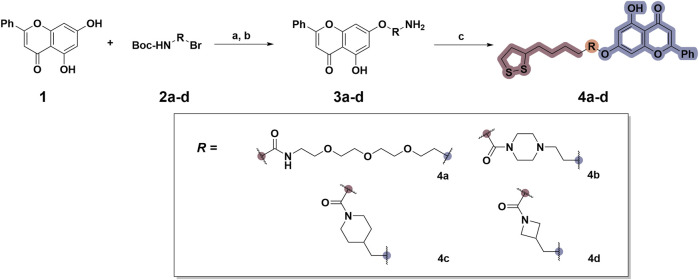
Synthetic routes for the synthesis of chrysin derivatives **4a–d** utilizing α-lipoic acid as a precursor. Regents and conditions: (a) K_2_CO_3_, KI, acetone, 65°C; (b) CF_3_COOH, DCM, r. t.; (c) HATU, DIPEA, DMF, α-LA, r. t.

**FIGURE 1:**
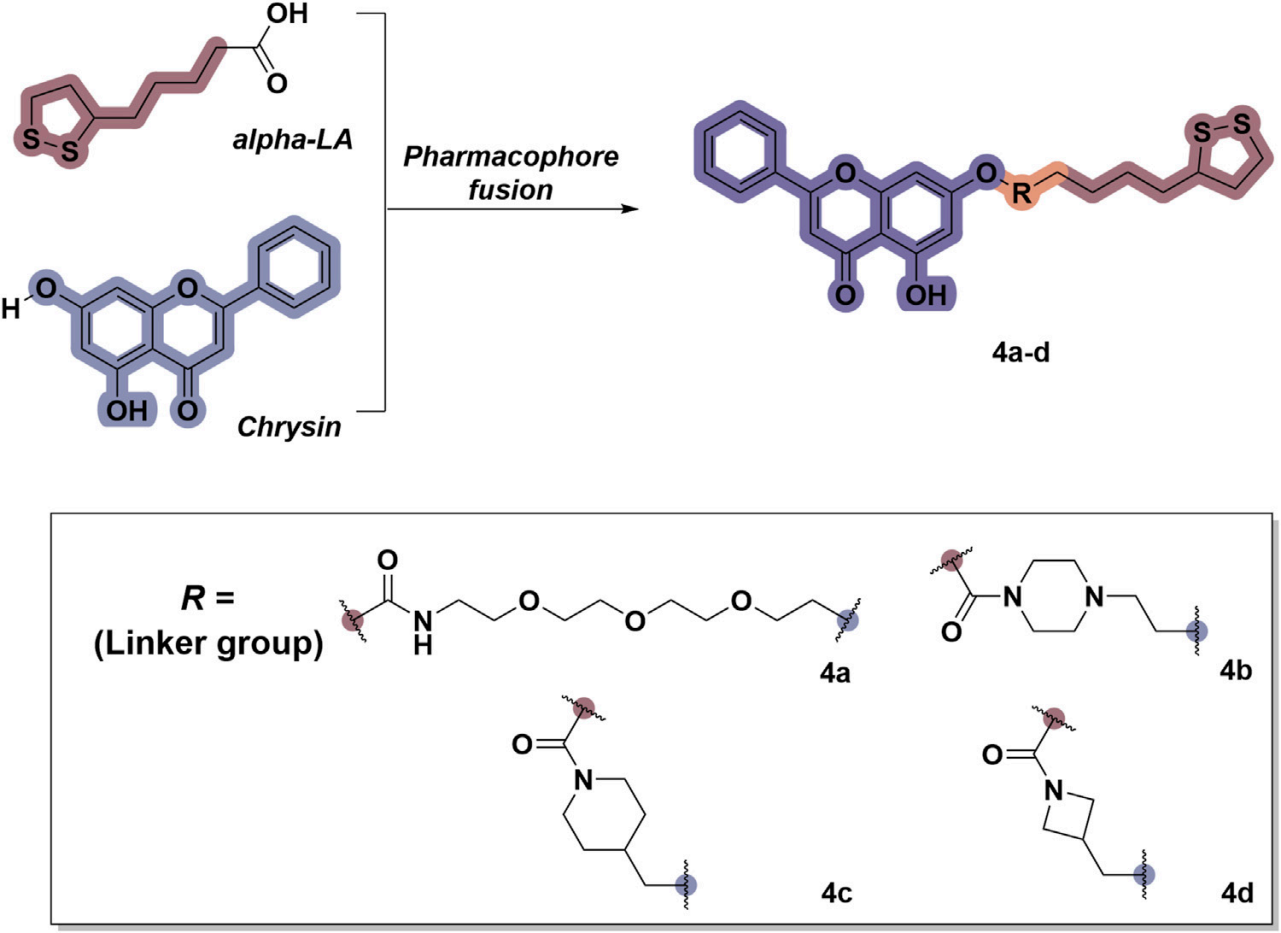
Design and synthesis of novel chrysin derivatives containing α-LA.

To date, several studies have demonstrated that the introduction of suitable substituents on the hydroxyl group at seven-position of chrysin could improve bioactivities. As demonstrated in Figure 1, this study involved the identification of a series of chrysin derivatives that integrate α-LA through a pharmacophore fusion strategy, which entailed the incorporation of α-LA into chrysin using diverse linker groups. Compounds **4a–d** can be prepared using the general procedure shown in Scheme 1. Specifically, chrysin **one** underwent a substitution reaction in the presence of K_2_CO_3_, potassium iodide, and compounds **2a–d** containing bromine atom and Boc-protected amino group to attach the intermediate. The deprotection of the compound was conducted utilizing trifluoroacetic acid in dichloromethane, followed by the coupling with 2-chloroethanesulfonyl chloride to yield compounds **3a–d**. Finally, compounds **4a–d** were synthesized by amide condensation reaction of **3a–d** and α-LA.

### 2.2 Biological evaluation

#### 2.2.1 Structure-activity relationship of synthesized molecules

TNF-α, a prominent cytokine, serves as a key mediator in the inflammatory response by facilitating the recruitment of white blood cells to the mucosa (Zhang L et al., 2019). Its pivotal role in initiating intestinal inflammation, a hallmark of IBD, underscores its significance in the pathogenesis of this condition (Larabi A et al., 2020). During the *in vitro* screening process aimed at identifying potential compounds with the ability to reduce intestinal inflammation, we assessed the inhibitory effects of all synthesized compounds (**4a–d**) on the adhesion of monocytes to HT-29 human colonic epithelial cells induced by TNF-α. Moreover, chrysin and α-LA were employed as standard reference compounds in the assay. As indicated in Table 1, the synthesized compounds **4a–d** exhibited significantly higher inhibitory activities in comparison to positive control molecules, with IC_50_ values falling within the low micromolar range. Furthermore, in comparison to compound **4a** containing a polyethylene glycol linker group, compounds **4b–d** incorporating rigid (cycloaliphatic) linker groups demonstrated increased effectiveness in reducing the TNF-α-induced adhesion of monocytic cells to colonic epithelial cells. This effect is likely attributed to the incorporation of a rigid linker group, which enhances the equilibrium between *in vitro* potency and physicochemical properties, thereby promoting cellular penetration. Among the synthesized molecules analyzed, compound **4b**, which contains a piperazine group, demonstrated the most potent inhibitory activity, as indicated by its IC_50_ value of 4.71 μM. Thus, This could potentially serve as a valuable initial step in the process of identifying a chrysin-based molecule that could be efficacious in the treatment of IBD.

**TABLE 1:** *In vitro* inhibitory potency of compounds **4a–d** against TNF-α-induced adhesion of monocytes to colon epithelial cells HT-29.

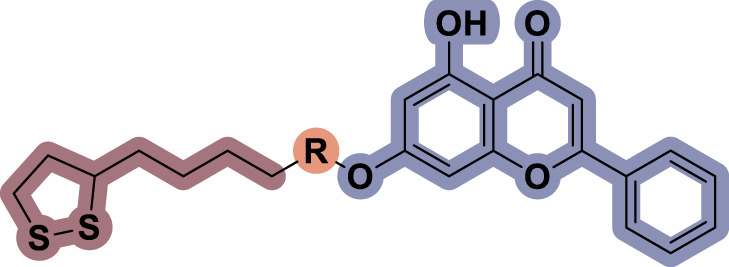		
Compd	R	IC_50_^a^ (μM)
**4a**	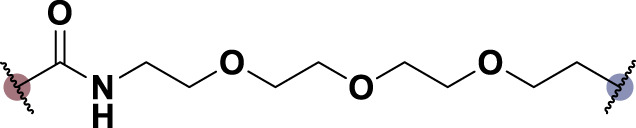	9.47 ± 0.48
**4b**	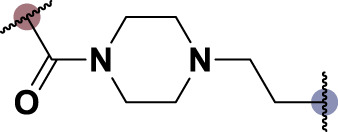	4.71 ± 0.16
**4c**	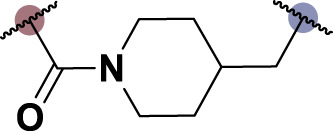	6.73 ± 0.94
**4days**	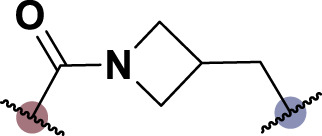	5.61 ± 0.32
Chrysin	–	>30
α-LA	–	12.7 ± 0.6

^a^ The results represent the mean ± standard deviation (SD) of three independent experiments conducted in triplicate.

#### 2.2.2 Compound 4b suppresses the adhesion induced by TNF-α in HT-29 cells by downregulating the expression of chemokine molecules and inhibiting the associated signaling pathways

**FIGURE 2:**
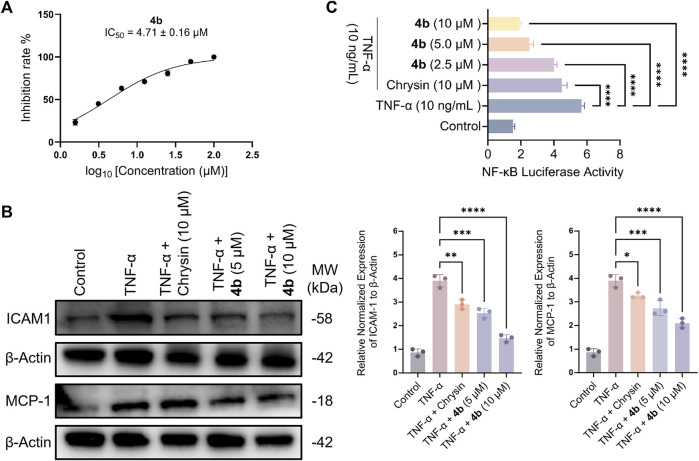
Compound **4b** demonstrates an *in vitro* anti-inflammatory effect. **(A)** The impact of **4b** on the adherence of monocytes to HT-29 cells stimulated by TNF-α was evaluated. Cell viability was assessed through the utilization of the cell-counting kit-8 (CCK-8) assay, which quantifies cell numbers. **(B)** Western blotting assay. The study examined the inhibitory impact of chrysin and **4b** on the expression of ICAM-1 and MCP-1 in TNF-α-stimulated HT-29 cells. β-Actin was utilized as a reference protein for the purpose of normalization. **(C)** The test compounds demonstrated inhibition of NF-κB transcriptional activity induced by TNF-α. The error bar displayed the SD, *p < 0.05, **p < 0.01, ***p < 0.001 and ****p < 0.0001, compared with the control groups.

As demonstrated in Figure 2A, compound 4b exhibited significant inhibitory activity against the adhesion of monocytes to HT-29 cells induced by TNF-α, with an IC_50_ value of 4.71 μM. Subsequently, this study investigated the potential mechanism of action of 4b by evaluating its influence on TNF-α-stimulated monocyte-epithelial adhesion in HT-29 cells, with a particular focus on determining if the inhibitory effect of 4b is due to the reduction of adhesion molecule expression. Figure 2B demonstrates that 4b effectively suppressed TNF-α-induced intercellular adhesion molecule (ICAM)-1 expression in a manner that was dependent on concentration. Numerous studies have demonstrated that TNF-α stimulates the expression of monocyte chemotactic protein-1 (MCP-1), a chemokine that plays a crucial role in directing the migration of leukocytes to sites of inflammation (Radaei Z et al., 2020; Navaei-Alipour N et al., 2021). Furthermore, **4b** significantly suppressed the expression of MCP-1 in a concentration-dependent manner.

Upon interaction with its receptors, TNF-α triggers a cascade of signaling pathways that ultimately culminate in the activation of nuclear factor-kappaB (NF-κB), a pivotal transcription factor involved in the regulation of genes associated with inflammatory processes (Wang Y. et al., 2023). In order to investigate the potential relationship between the inhibitory effect of **4b** on ICAM-1 and MCP-1 expression, as well as its impact on NF-κB transcriptional activity, the inhibitory potency of **4b** on NF-κB transcription was determined. The observed inhibition of NF-κB activity in HT-29 cells by **4b** exhibited a dose-dependent effect, as illustrated in Figure 2C.

#### 2.2.3 ROS production induced by TNF-α is effectively inhibited by compound 4b

**FIGURE 3:**
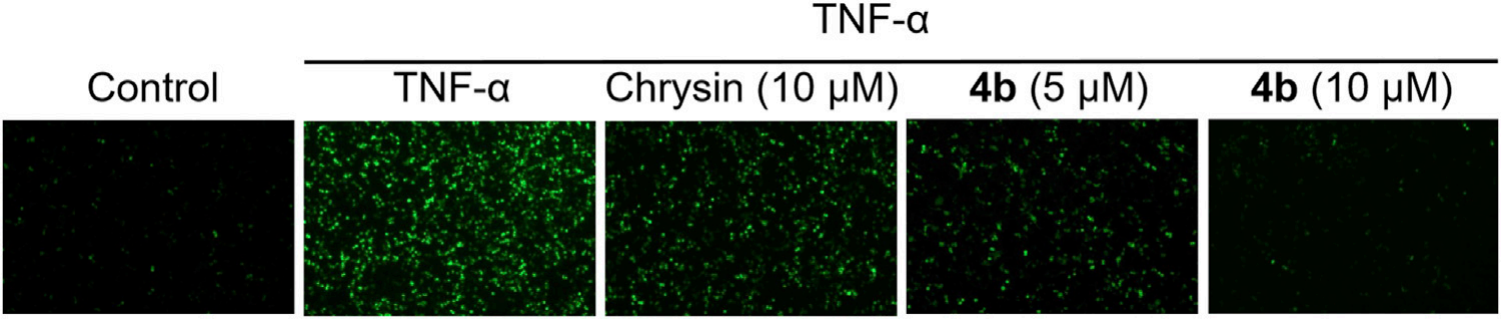
Inhibition of ROS production induced by TNF-α through the application of **4b**. The HT-29 cells were subjected to pretreatment with test compounds for a duration of 1 h before being exposed to TNF-α (10 ng/mL) for a period of 30 min. The cells were subjected to treatment as outlined in the ROS assay kit protocol. Fluorescence microscopy was employed to observe intracellular ROS.

The activation of NF-κB in the downstream signaling of TNF-α is dependent on reactive oxygen species (ROS), which are regulated by redox-sensitive transcription factors (Thoma and Lightfoot, 2018). This study further examined the ability of compound **4b** to inhibit the production of ROS induced by TNF-α. Firstly, the ROS assay kit was utilized to identify ROS, and TNF-α-induced HT-29 cells were exposed to varying concentrations of test compounds for a duration of 48 h. Subsequently, the cells were treated in accordance with the manufacturer’s instructions of the kit, and the levels of ROS were quantified using fluorescence microscopy. As illustrated in Figure 3, compound **4b** demonstrated a notable inhibitory effect on the generation of ROS induced by TNF-α in a dose-dependent fashion. These findings are congruent with the outcomes obtained through flow cytometry analysis.

#### 2.2.4 The advantageous effects of compound 4b in mitigating TNBS-induced colitis in rat models

**FIGURE 4:**
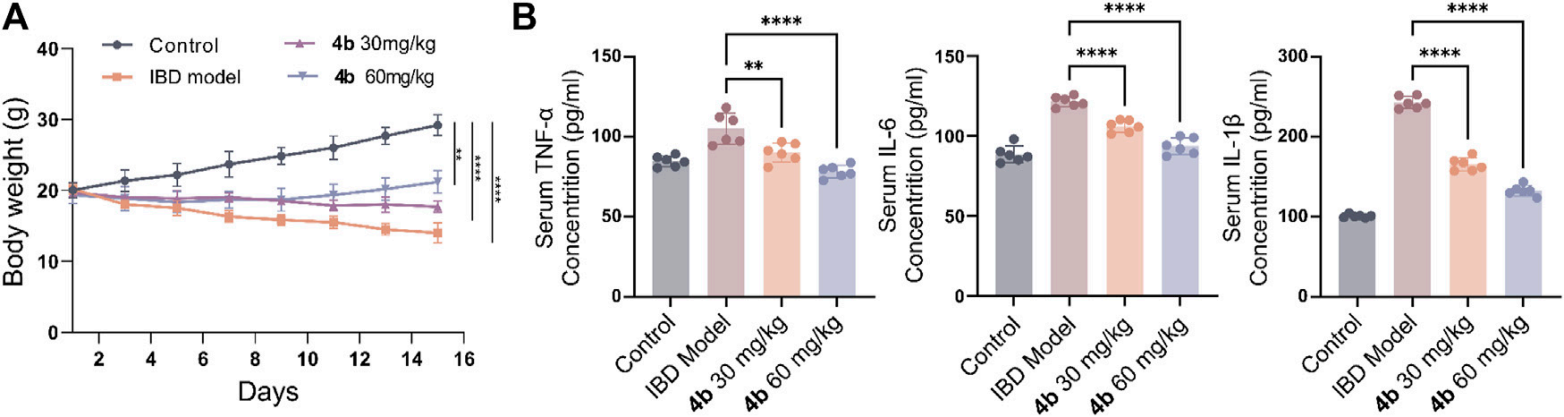
The advantageous effects of compound **4b** in mitigating TNBS-induced colitis in rat models. **(A)** Changes in body weight in rats with colitis induced by TNBS after treatment with the experimental compound. **(B)** The levels of pro-inflammatory cytokines, specifically TNF-α, IL-1β, and IL-6, in serum were measured *via* ELISA to assess the suppressive impact of **4b** on their production. The error bar indicated the SD, **p < 0.01 and ***p < 0.001 compared with the control groups.

**FIGURE 5:**
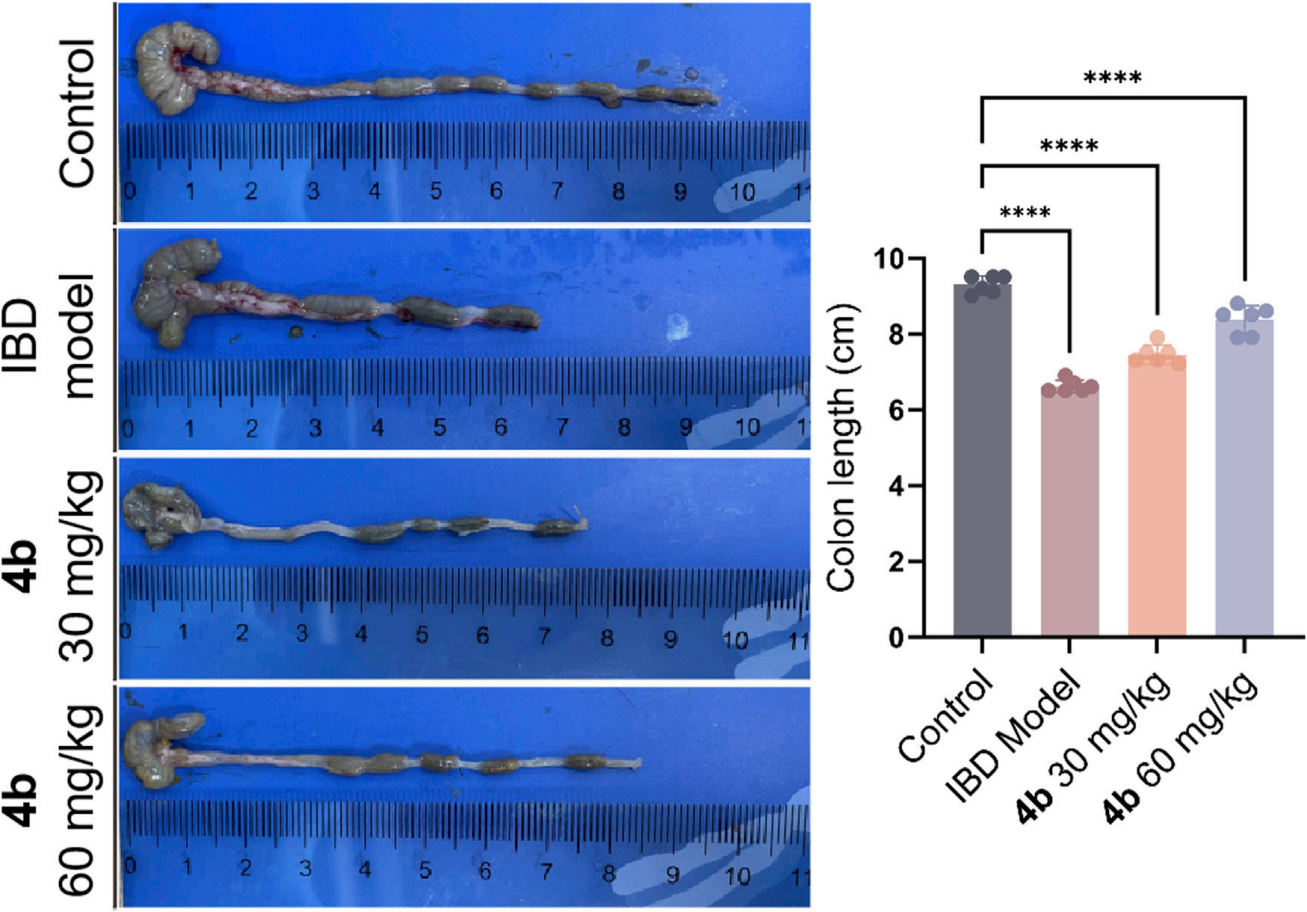
The positive effects of compound **4b** in mitigating TNBS-induced colitis in rat models. The length of the colon was measured following dissection. The error bar indicated the SD, ****p < 0.0001, compared with the control groups.

**FIGURE 6:**
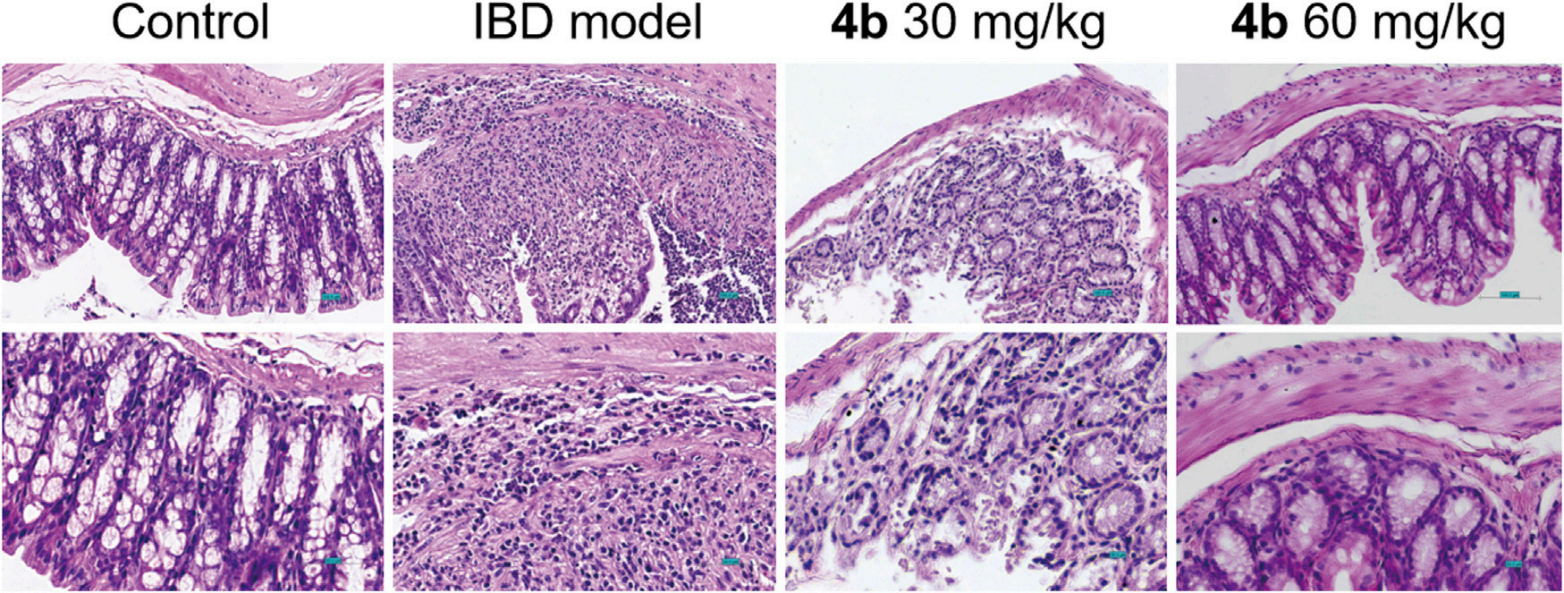
Representative H&E staining of colon sections from rat models with TNBS-induced colitis treated with **4b**. Scale bar = 100 μM.

The efficacy of **4b** in treating IBD was assessed in a rat model of colitis induced by 2, 4, 6-trinitro-benzenesulfonic acid (TNBS). Next, the rats received oral administration of **4b** at dosages of either 30 or 60 mg/kg. Rats subjected to TNBS colitis displayed manifestations of inflammation, hematochezia, weight loss, and decreased mobility relative to the control group. Additionally, TNBS-treated rats exhibited a substantial decline in body weight, stunted growth, and a notable increase in colon tissue weight attributed to congested edema (Figure 4A). The TNBS-induced colitis was effectively mitigated in a dose-dependent manner following oral administration of **4b**. Significantly, notable improvements were observed in terms of both body weight loss and inflammation in the colon tissue. The administration of 60 mg/kg **4b** demonstrated the most significant efficacy in the treatment of TNBS-induced colitis (Figure 5). Furthermore, in order to investigate the impact of 4b on the reversal of mucosal inflammation and damage, histological examination using hematoxylin and eosin (H&E) staining was conducted on colonic tissue sections from various experimental groups. The results depicted in Figure 6 demonstrate that treatment with 4b significantly reduced colonic inflammation and crypt damage induced by TNBS in mice.

**FIGURE 7:**
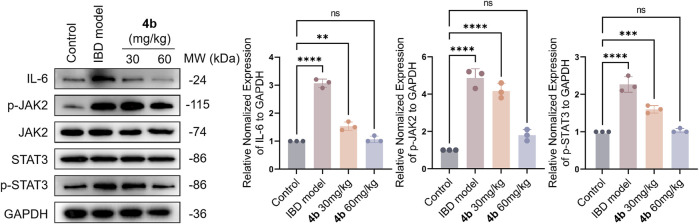
Western blotting assay. The impact of test compound on the modulation of TNF-α, p-JAK2, total JAK2, p-STAT3, and total STAT3 in colonic tissues was assessed, with the expression of GAPDH serving as an internal control. ns, not significant. The error bar indicated the SD, **p < 0.01, ***p < 0.001 and ****p < 0.0001, compared with the control groups.

The secretion of pro-inflammatory mediators is a characteristic feature of colitis induced by TNBS. Our findings in Figure 4B indicate a significant increase in the levels of IL-1β, IL-6, and TNF-α in the serum following TNBS challenge. Importantly, **4b** demonstrated a dose-dependent inhibition of the production of these pro-inflammatory cytokines. Furthermore, the JAK/STAT signaling pathway plays a significant role in inflammatory processes, as evidenced by numerous studies indicating that cytokine signaling is initiated through activation of the JAK and STAT family of kinases. In order to clarify the precise mechanism underlying the therapeutic effects of **4b** *in vivo*, we conducted an analysis of various inflammation-related markers through Western blotting assay, including the pro-inflammatory cytokine IL-6, phosphorylated JAK2 (p-JAK2), total JAK2, phosphorylated STAT3 (p-STAT3), and total STAT3. The data presented in Figure 7 indicates a significant increase in the levels of IL-6, p-JAK2, and p-STAT3 in the colonic tissues of mice with TNBS-induced colitis. Furthermore, in comparison to the TNBS-induced group, **4b** demonstrated the ability to inhibit the expression of IL-6, p-JAK2, and p-STAT3 in a dose-dependent manner. Notably, the findings demonstrate that **4b** did not alter the overall levels of JAK2 and STAT3 in colonic tissues across all concentrations tested, indicating that the inhibition of p-JAK2 and p-STAT3 was not influenced by potential cytotoxic effects of the compound. Collectively, this data indicates that **4b** possesses healing properties for IBD and may serve as a novel candidate for IBD treatment.

#### 2.2.5 ADMET profile of synthesized compounds

To assess the drug-likeness and pharmacokinetic characteristics of the recently developed compounds, an *in silico* ADMET screening was conducted. The results, presented in Supplementary Table S1, include an evaluation of various factors such as molecular weight, hydrogen-bond acceptor/donor count, blood-brain barrier permeability, and drug-likeness. The anticipated outcomes indicated enhancements in the physical and chemical characteristics of the synthesized compound in comparison to chrysin. However, despite the favorable safety profile of the synthesized compounds, their absorption, distribution, and metabolic data are suboptimal. Additional investigations into the structure-activity relationship of this series of molecular structures are warranted to identify promising derivatives with favorable ADMET properties for the management of IBD.

## 3 Experimental

### 3.1 Chemistry

Commercially available reagents and solvents were utilized without any additional purification. Column chromatography was performed using silica gel (100–200 mesh) as the medium for purification purposes. A fluorescent indicator was employed for real-time monitoring of the reaction, while UV light at wavelengths of 254 and 365 nM was utilized to visualize the markings on silica gel plates. A Bruker AV-600 spectrometer (^1^H, 400 MHz; ^13^C, 101 MHz) was utilized for the measurement of nuclear magnetic resonance (NMR) spectra, employing tetramethylsilane (TMS) as the internal reference compound. In NMR spectra analysis, spin multiplicities are denoted using the subsequent abbreviations. The values of coupling constants (*J*) are expressed in hertz units (Hz). Proton coupling patterns were denoted as singlet (s), doublet (d), triplet (t), quartet (q), multiplet (m), and doublet of doublets (dd). In reference to TMS, chemical shifts were reported using parts per million notation (ppm, *δ*). The test compounds were determined to have a purity exceeding 95% using an analytical high-performance liquid chromatography (HPLC) instrument (Agilent, Santa Clara, CA, United States). A GL-C18 reverse phase column (250 mm × 4.6 mm ×5 μM) was employed with ultra-pure water and methanol (chromatographic grade) as the mobile phase prior to assessing their biological activities. The HRMS analysis was carried out employing Agilent LC/MSD TOF mass spectrometers. Melting point was obtained by melting point apparatus (SGWX-4, Shanghai ShenGuang Instrument Co., Ltd, Shanghai, China).

General procedure for the synthesis of **4a–d** 5-[1, 2-Dithiolan-3-yl)-N-(2-(2-(2-(2-((5-hydroxy-4-oxo-2-phenyl-4H-chromen-7-yl)oxy)ethoxy)ethoxy)ethoxy)ethyl)pentanamide (**4a**).

Step 1: Chyrisn (1 mmol) was introduced into a vigorously stirred mixture containing tert-butyl (2-(2-(2-(2-bromoethoxy)ethoxy)ethoxy)ethyl)carbamate **2a** (1.05 mmol), K_2_CO_3_ (2 mmol), and KI (0.1 mmol) dissolved in acetone (20 mL). The reaction mixture underwent stirring and reflux at a temperature of 65 °C for a duration of 16 h. Following the conclusion of the reaction, the mixture was cooled to ambient temperature. Subsequent filtration was conducted, and the resultant filtrate underwent further concentration and purification via column chromatography on silica gel utilizing a petroleum ether and ethyl acetate mixture in a 15:1 ratio. This procedure resulted in the formation of a yellow solid intermediate. Subsequently, the compound was dissolved in 5 mL of dichloromethane and subsequently treated with 2.5 mL of trifluoroacetic acid. The resulting mixture was stirred for 6 h at room temperature. To neutralize the reaction solution, saturated aqueous sodium bicarbonate was employed, followed by extraction with three portions of dichloromethane, each consisting of 30 mL. After being separated, the organic layer was washed with a saturated sodium chloride solution, dried with anhydrous Na_2_SO_4_, and evaporated, leading to the production of a yellow solid. This solid was utilized in the subsequent reaction without further purification steps.

Step 2: A solution comprising compound **3a** (0.25 mmol), HATU (0.25 mmol), α-LA (0.27 mmol), and DIPEA (0.5 mmol) in 30 mL of DMF was subjected to gentle stirring at room temperature for 20 h. Upon completion of the reaction, the resulting mixture was quenched by addition to 150 mL of ice-cold water, leading to the formation of a solid product upon filtration. The crude product was purified via silica gel chromatography utilizing a solvent system composed of methylene chloride and methanol in a 30:1 ratio, resulting in the isolation of pale yellow solid **4a** (0.49 g, 32% yield). mp 223.5°C–225.4°C. ^1^H NMR (400 MHz, DMSO-*d*_*6*_) *δ* 12.72 (s, 1H), 7.95–7.80 (m, 3H), 7.56–7.47 (m, 3H), 6.83 (s, 1H), 6.59 (s, 1H), 6.25 (s, 1H), 4.13 (s, 2H), 3.62–3.40 (m, 11H), 3.26–3.20 (m, 2H), 3.13—3.00 (m, 2H), 2.39–2.29 (m, 1H), 2.07 (t, *J* = 7.3 Hz, 2H), 1.84–1.73 (m, 1H), 1.64–1.43 (m, 5H), 1.34–1.24 (m, 2H). ^13^C NMR (101 MHz, DMSO-*d*_*6*_) *δ* 182.25, 172.65, 172.60, 164.79, 163.65, 161.62, 157.55, 132.33, 130.93, 129.37, 126.65, 126.60, 105.60, 105.31, 98.75, 93.37, 70.88, 70.45, 70.09, 70.04, 69.73, 69.06, 68.44, 56.59, 38.64, 38.52, 35.62, 34.59, 32.46, 28.81, 25.52. HRMS (ESI) (*m/z*): [M + H]^+^ calcd for C_31_H_40_NO_8_S_2_ 618.2190, found 618.2197. Purity: 98.00% (HPLC, t_R_ = 11.13 min).

7-(2-(4-(5-(1,2-Dithiolan-3-yl)pentanoyl)piperazin-1-yl)ethoxy)-5-hydroxy-2-phenyl-4H-chromen-4-one (**4b**) yellow solid (0.20 g, 15% yield); mp 194.7°C–197.0°C. ^1^H NMR (400 MHz, Chloroform-*d*) *δ* 12.66 (s, 1H), 7.79 (d, *J* = 7.3°Hz, 2H), 7.52–7.45 (m, 3H), 6.56 (s, 1H), 6.41 (s, 1H), 6.25 (d, *J* = 1.9 Hz, 1H), 4.12 (t, *J* = 5.5°Hz, 2H), 3.64 (t, *J* = 4.7 Hz, 2H), 3.49 (d, *J* = 5.1 Hz, 2H), 3.22—3.01 (m, 2H), 2.88—2.73 (m, 5H), 2.59–2.52 (m, 4H), 2.47–2.39 (m, 1H), 2.33 (t, *J* = 7.5 Hz, 2H), 1.93–1.85 (m, 1H), 1.66–1.59 (m, 2H), 1.54—1.38 (m, 2H). ^13^C NMR (101 MHz, Chloroform-*d*) *δ* 181.83, 170.78, 164.26, 163.33, 161.62, 157.19, 131.63, 130.67, 128.80, 125.91, 105.24, 98.38, 92.69, 66.29, 56.43, 56.26, 53.51, 53.06, 45.27, 41.27, 40.03, 38.41, 38.33, 36.22, 34.56, 32.65, 31.14, 28.85, 24.80. HRMS (ESI) (*m/z*): [M + H]^+^ calcd for C_29_H_35_N_2_O_5_S_2_ 555.1982, found 555.1982. Purity: 99.36% (HPLC, t_R_ = 11.84 min).

7-((1-(5-(1,2-Dithiolan-3-yl)pentanoyl)piperidin-4-yl)methoxy)-5-hydroxy-2-phenyl-4H-chromen-4-one (**4c**) yellow solid (0.33 g, 28% yield); mp 189.0°C–191.5°C. ^1^H NMR (400 MHz, Chloroform-*d*) *δ* 12.70 (s, 1H), 7.95—7.78 (m, 2H), 7.57–7.49 (m, 3H), 6.65 (s, 1H), 6.50 (d, *J* = 2.3 Hz, 1H), 6.35 (d, *J* = 2.1 Hz, 1H), 6.14 (d, *J* = 5.8 Hz, 1H), 4.19 (dd, *J* = 5.6, 3.3 Hz, 2H), 3.85 (dd, *J* = 5.6, 3.3 Hz, 2H), 3.64 (t, *J* = 5.0 Hz, 2H), 3.58—3.45 (m, 4H), 3.19—3.03 (m, 2H), 2.81 (s, 1H), 2.45–2.38 (m, 1H), 2.19 (t, *J* = 7.5 Hz, 2H), 1.91–1.83 (m, 2H), 1.68—1.59 (m, 3H), 1.52—1.34 (m, 2H). ^13^C NMR (101 MHz, Chloroform-*d*) *δ* 182.37, 172.96, 164.63, 164.03, 162.06, 157.64, 131.94, 131.11, 129.11(2), 126.25(2), 105.75, 98.61, 93.18, 70.14, 69.08, 67.92, 56.43, 40.22 (2), 39.14, 38.45, 36.38, 34.60 (2), 28.88, 25.40 (2). HRMS (ESI) (*m/z*): [M + H]^+^ calcd for C_29_H_34_NO_5_S_2_ 540.1890, found 540.1946. Purity: 99.36% (HPLC, t_R_ = 11.84 min).

7-[(1-(5-(1,2-Dithiolan-3-yl)pentanoyl)azetidin-3-yl)methoxy)-5-hydroxy-2-phenyl-4H-chromen-4-one (**4d**ays)]

Yellow solid (0.26 g, 19% yield); mp 176.7°C–178.9°C. ^1^H NMR (400 MHz, Chloroform-*d*) *δ* 12.67 (s, 1H), 7.78 (t, *J* = 5.6 Hz, 2H), 7.48 (dd, *J* = 12.5, 7.3 Hz, 3H), 6.55 (d, *J* = 4.3 Hz, 1H), 6.42 (s, 1H), 6.25 (t, *J* = 2.6 Hz, 1H), 4.29 (t, *J* = 8.5 Hz, 1H), 4.20—3.97 (m, 4H), 3.87 (dd, *J* = 10.1, 5.2 Hz, 1H), 3.66—3.50 (m, 1H), 3.18–3.03 (m, 3H), 2.48–2.39 (m, 1H), 2.12 (t, *J* = 7.2 Hz, 2H), 1.94–1.85 (m, 1H), 1.74–1.64 (m, 4H), 1.55—1.39 (m, 2H). ^13^C NMR (101 MHz, CDCl_3_) *δ* 182.18, 173.08, 164.33, 163.81, 162.00, 157.48, 131.87, 130.90, 129.02(2), 126.13(2), 105.57, 98.47, 92.97, 92.94, 69.38, 56.40, 52.47, 49.81, 40.20, 38.48, 34.65, 30.98, 28.97, 27.73, 24.49. HRMS (ESI) (*m/z*): [M + Na]^+^ calcd for C_27_H_29_NNaO_5_S_2_ 534.1379, found 534.1388. Purity: 100% (HPLC, t_R_ = 10.63 min).

### 3.2 Biology

#### 3.2.1 Cell culture and antibodies

The HT29 and U937 cell lines, representing a human colonic epithelial cell line and a pre-monocytic cell line respectively, were acquired from the Shanghai Cell Bank of the Chinese Academy of Science (Shanghai, China). The cells were cultured in RMPI 1640 media supplemented with 10% fetal bovine serum and 1% Penicillin/Streptomycin. The incubation was carried out at a temperature of 37°C in a CO_2_-humidified incubator with a concentration of 5%.

The following antibodies were used in this study: GAPDH (CST, no. 2118), IL-6 (CST, no. 12912), JAK2 (CST, no. 3230), Phospho-JAK2 (CST, no. 3771), STAT3 (CST, no. 12640), Phospho-JAK2 (CST, no. 9145), TNF-α (CST, no. 11948), ICAM-1 (CST, no. 67836S), MCP-1 (CST, no. 2029), and E-cadherin (CST, no. 3195).

#### 3.2.2 TNF-α-stimulated adhesion of monocytic cells to colonic epithelial cells

A previously established method was employed to conduct the adhesion assay, utilizing a cultured monolayer of HT29 cells and non-adherent monocytic cell U937 cells. The adhesion of U937 monocytic cells to colonic epithelial cells was assessed by utilizing human U937 pre-monocytic cells. These cells were previously labeled with BCECF/AM (10 μg/mL) for 1 h at a temperature of 37°C. The HT-29 cells were cultured in 24-well plates and treated with the test compound for 60 min before being exposed to TNF-α (10 ng/mL) and IL-6 (5 ng/mL) for an additional 180 min. Following this, the cells were co-incubated with U937 cells that had been prelabeled with BCECF/AM (1 × 10^6^ cells/well) for a duration of 30 min at a temperature of 37°C. The U937 cells that did not adhere were eliminated, while the HT-29 cells and U937 cells that adhered were rinsed twice with PBS. For quantitative analysis, additional cell samples were subjected to lysis using a solution containing 0.1% Triton X-100 in Tris (0.1 M). The resulting fluorescence was then measured utilizing a fluorescence-detecting microplate reader (Synergy MX, Biotek) with excitation and emission wavelengths set at 580 nm.

#### 3.2.3 NF-κB reporter assay

To assess the activation of NF-κB, HT-29 cells were subjected to transfection with an NF-κB reporter gene utilizing the CignalTM NF-κB Reporter luciferase Kit (Qiagen Ltd., Manchester, UK). Initially, the cells were seeded onto a 24-well culture plate in a medium supplemented with 10% fetal bovine serum and devoid of antibiotics. Following a 24-h incubation period, the cells were transfected with the constructs utilizing Lipofectamine 2000 (Invitrogen, United States) in accordance with the manufacturer’s guidelines. Following a 24-h period, the transfection media was substituted with RPMI 1640 supplemented with 10% fetal bovine serum, allowing the cells an additional day for proliferation. Prior to subjecting the transfected cells to TNF-α treatment for a duration of 3 h, they were pre-treated with various compounds for 1 hour. Subsequently, TNF-α was administered and incubated for another 3 hours. The cell lysates were obtained using a lysis buffer and analyzed following the manufacturer’s instructions.

#### 3.2.4 TNBS-stimulated colitis

The animal experiments conducted in this study were approved by the relevant committee at Chengdu University of Traditional Chinese Medicine and were carried out in accordance with institutional guidelines for animal research (ethical review number: 20231012). Female Sprague-Dawley (SD) rats were utilized to assess the *in vivo* anti-IBD activity of the test compounds. Experimental colitis was induced using 2, 4, 6-trinitrobenzenesulfonic acid (TNBS) as previously described. Prior to the administration of TNBS, rats underwent a 24-h fasting period and were lightly anesthetized with diethyl ether. Subsequently, a solution containing 1.0 mL of TNBS at a concentration of 5% was gently introduced into the colon, approximately 7 cm away from the anus. This is achieved by affixing a polyethylene catheter to a 1 mL syringe. In contrast, the animal models in the control group underwent a similar procedure but were administered with ethanol at a concentration of 50% instead. Following the administration of TNBS, the rats were placed in an upright position for a duration of 60 s before being returned to their enclosure. Compound **4** **d**ays, at doses of 30 or 60 mg/kg/day and suspended in a solution of 10% DMSO, 15% sulfobutylether-β-cyclodextrin, and 75% saline, was orally administered on the day of TNBS administration. On the 15th day of the experiment, the rats were euthanized, and the severity of colitis and visible ulcers in the mice was assessed by trained professionals. The colon tissue located 6–9 cm proximal to the rectum was surgically removed, followed by analysis of myeloperoxidase content and histological examination.

#### 3.2.5 Western blotting

The rat colon tissue, weighing 45 mg, was pulverized and homogenized in 1X PBS using the Bead blaster from Benchmark Scientific. Following centrifugation of the homogenates, the lysate was resuspended in RIPA buffer supplemented with a cocktail of protease inhibitors and phosphatase inhibitors to extract the proteins. The BCA protein assay kit (Beyotime Biotechnology, Jiangsu, China) was utilized to quantify the concentration of protein samples. Nitrocellulose membranes were employed for transferring equivalent quantities of total protein resolved on SDS-PAGE gels. Afterwards, the membranes were obstructed using skim milk with a concentration of 5%. Subsequently, primary antibodies and their corresponding secondary antibodies were introduced to the membranes for incubation. Ultimately, the protein bands were made visible by utilizing the ECL chemiluminescent HRP substrate.

#### 3.2.6 Hematoxylin and eosin (H&E) staining

The colon samples were surgically removed, preserved in a 4% formaldehyde solution, encased in paraffin wax, and sliced into sections. After the removal of paraffin and restoration of moisture, longitudinal sections with a thickness of 5 μM were subjected to hematoxylin staining for a duration of 5 min. Subsequently, they underwent incubation in acid ethanol solution (1% HCl in 70% ethanol) followed by rinsing with distilled water. The sections were subsequently stained with eosin for 5 min, dehydrated using a graded series of alcohol solutions, and cleared in xylene. The slides that were mounted underwent examination, photography, and observation for any pathological alterations utilizing a digital bright-field microscope (BZ-9000, Keyence, Japan).

#### 3.2.7 Analysis of intracellular ROS production

As per the guidelines provided by the manufacturer, the ROS assay kit from Beyotime Biotechnology was utilized to assess the levels of intracellular ROS following treatment with the test compounds. Following this, intracellular ROS production was assessed using a fluorescence microscopy (MZ16FA, Leica, Germany). Statistical analysis was performed using GraphPrism software.

#### 3.2.8 Quantification of levels of cytokines associated with inflammation

Serum samples were obtained from mice blood through centrifugation at 4,000 *g* for 12 min, followed by measurement of serum cytokine levels using specific ELISA kits from Abcam (ab181421, ab178013, and ab214025).

#### 3.2.9 Statistical analysis

The validity of the findings was verified through replication in a minimum of three independent experiments. Data were presented as means ± standard deviation and subjected to statistical analysis using GraphPad Prism 8.0 software. Significance levels were determined by either one-way analysis of variance (ANOVA) or Student’s t-test, with a threshold of *p* < 0.05 indicating statistical significance.

#### 3.2.10 *In-silico* ADMET analysis

In this research, the utilization of ADMETlab facilitated the prediction of ADMET properties for synthesized molecules (https://admetmesh.scbdd.com). ADMETlab, an online computational tool, offers a variety of models for calculating molecular properties and pharmacokinetics, encompassing solubility, plasma protein binding, liver metabolism, renal excretion, among others. These models enable the prediction of drug absorption, distribution, metabolism, and excretion processes within the human body, as well as the assessment of potential toxicity and safety.

## 4 Conclusion

At present, natural products are great treasures for the identification of the novel lead molecules for IBD treatment. Although chrysin demonstrates a range of biological activities, studies have shown that the compound has limited water solubility and low bioavailability. Hence, the use of chrysin as a precursor for structural modification holds significant promise in the identification of potential therapeutic agents. In this study, the incorporation of the essential pharmacophore into α-lactalbumin has led to the identification of a series of novel chrysin derivatives derived from α-lactalbumin. These derivatives have shown promising efficacy in inhibiting the adhesion of monocytes to colon epithelium induced by TNF-α. Compound **4b** exhibited the highest inhibitory potency compared to the other compounds, with an IC_50_ value of 4.71 μM. *In vitro* experiments demonstrated that compound 4b effectively inhibited monocyte adhesion to epithelial cells, reduced ROS production induced by TNF-α, suppressed the levels of ICAM-1 and MCP-1, decreased NF-κB activity, and ameliorated TNBS-induced colitis in rat models. This study offers a novel approach for identifying chrysin-based compounds with potential therapeutic activity for the treatment of IBD.

## Data Availability

The original contributions presented in the study are included in the article/Supplementary Material, further inquiries can be directed to the corresponding authors.

## Abbreviations

α-LA, α-lipoic acid; CCK-8, cell-counting kit-8; H&E, hematoxylin and eosin; IBD, inflammatory bowel disease; ICAM, intercellular adhesion molecule; IFNγ, interferon gamma; iNOS, inducible nitric oxide synthase; LPS, lipopolysaccharide; MCP-1, monocyte chemotactic protein-1; NF-κB, nuclear factor-kappaB; NMR, nuclear magnetic resonance; NO, nitric oxide; ROS, reactive oxygen species; TMS, tetramethylsilane; TNBS, 2,4,6-trinitro-benzenesulfonic acid; SD, Sprague-Dawley.
