# Supplementary material for: Synthesis and biological evaluation of chrysin derivatives containing α-lipoic acid for the treatment of inflammatory bowel disease
Source: Front Chem. 2024 May 27;12:1406051. doi: 10.3389/fchem.2024.1406051 (PMC11163049; doi:10.3389/fchem.2024.1406051)
Supplement: Supplementary file 1 [file DataSheet1.DOCX]

**Support Information**

**Table of Contents**

1. **NMR spectra**....................................................................................................................S1
2. **Representative HPLC traces of biological tested compounds**.....................................S5
3. **HRMS spectra**..................................................................................................................S7
4. **Table S1 ADMET analysis of the synthesized compounds and chrysin**.......................S9

**
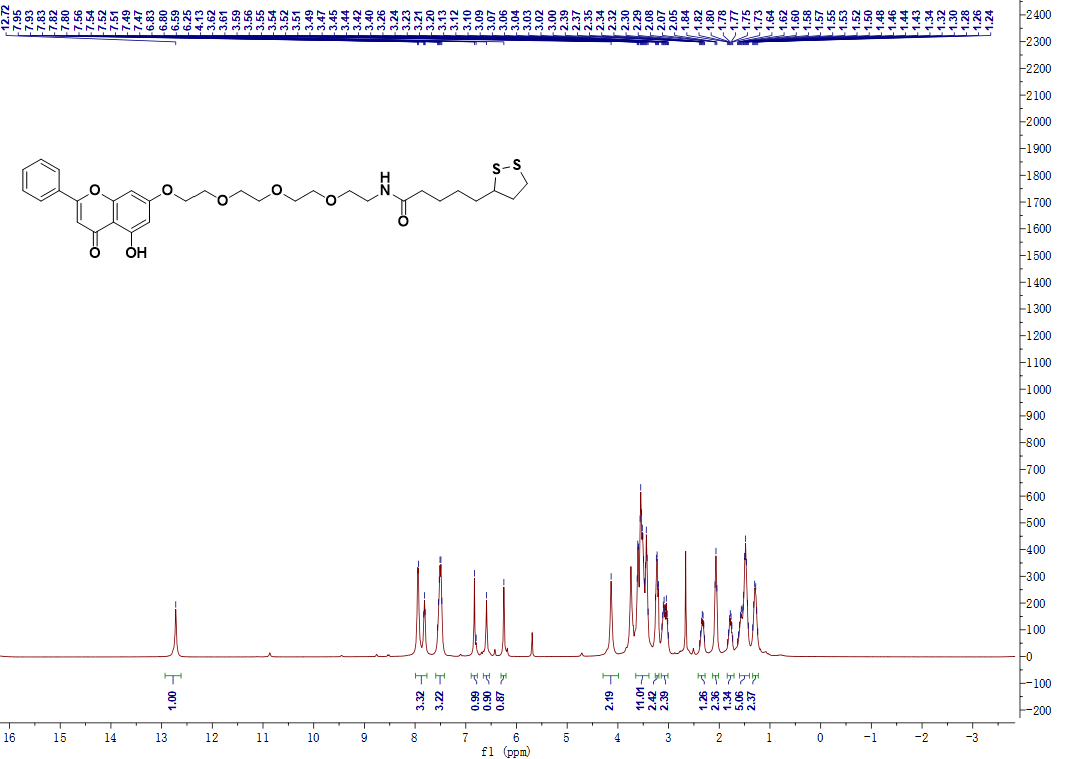
**

^1^H NMR Spectrum of Compound **4a**

**
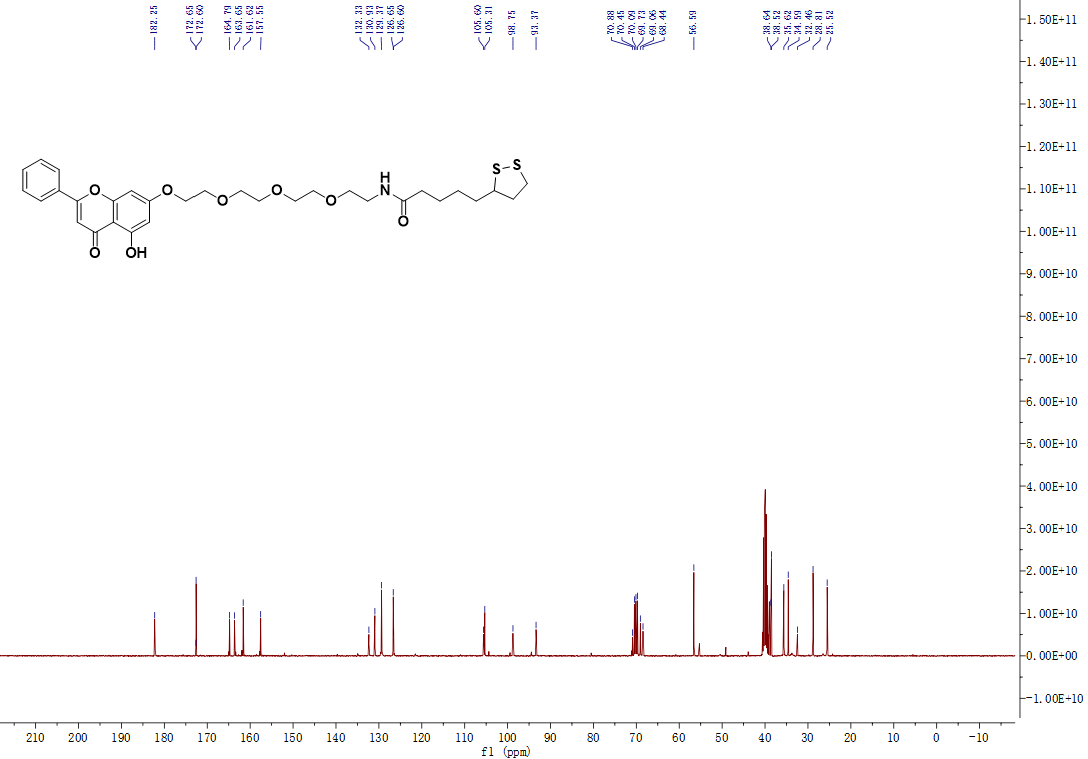
**

^13^C NMR Spectrum of Compound **4a**

**
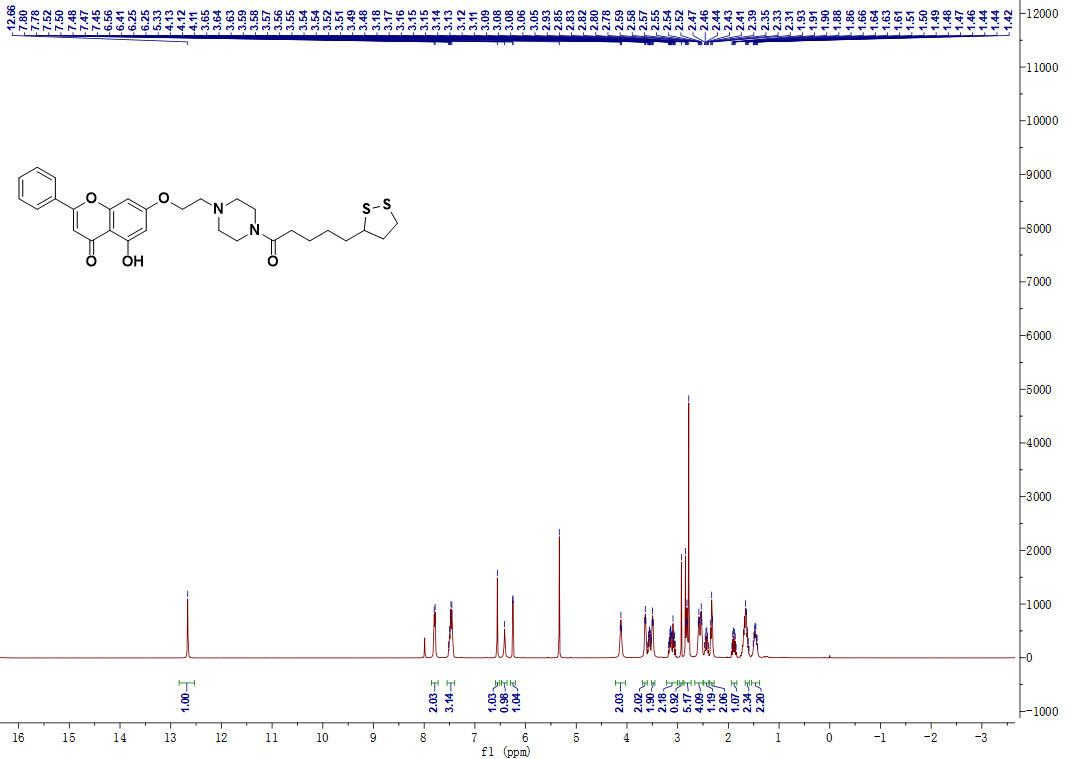
**

^1^H NMR Spectrum of Compound **4b**

**
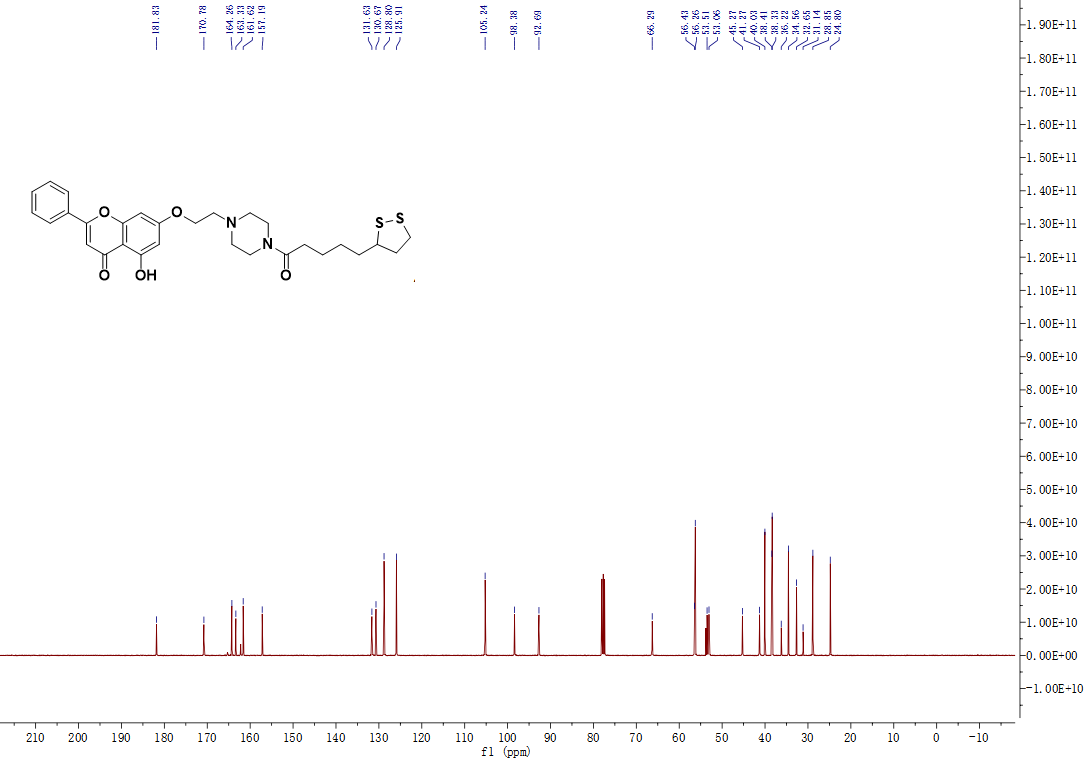
**

^13^C NMR Spectrum of Compound **4b**

**
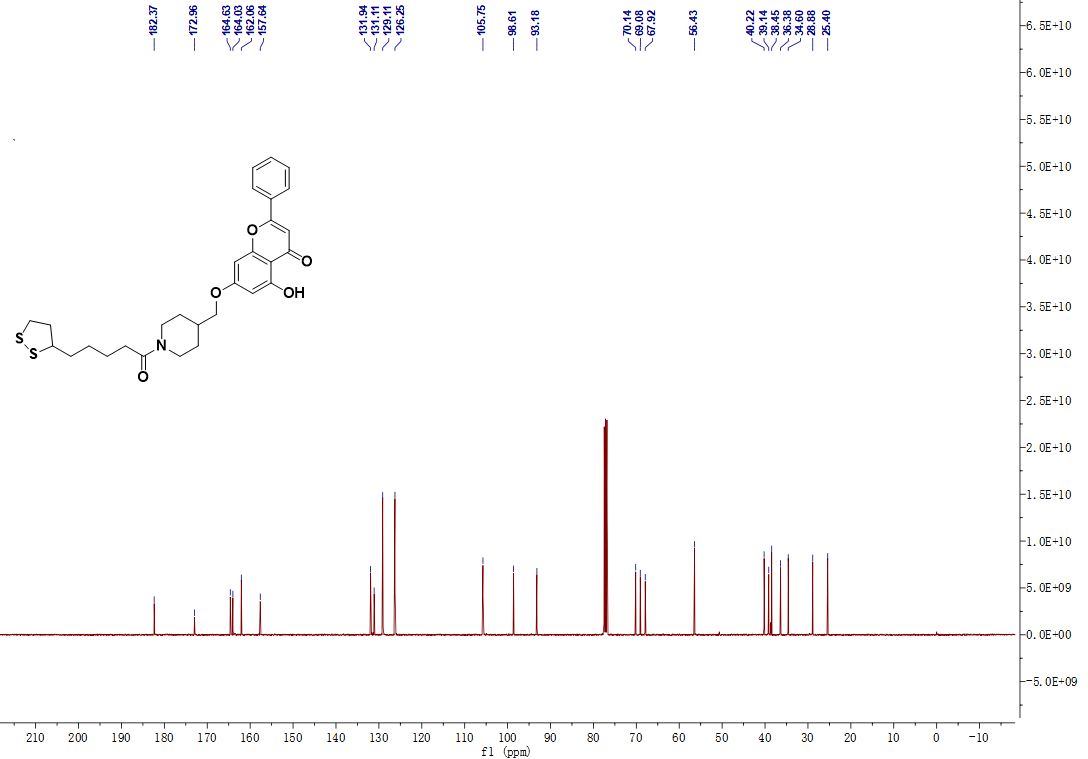
**

^1^H NMR Spectrum of Compound **4c**

**
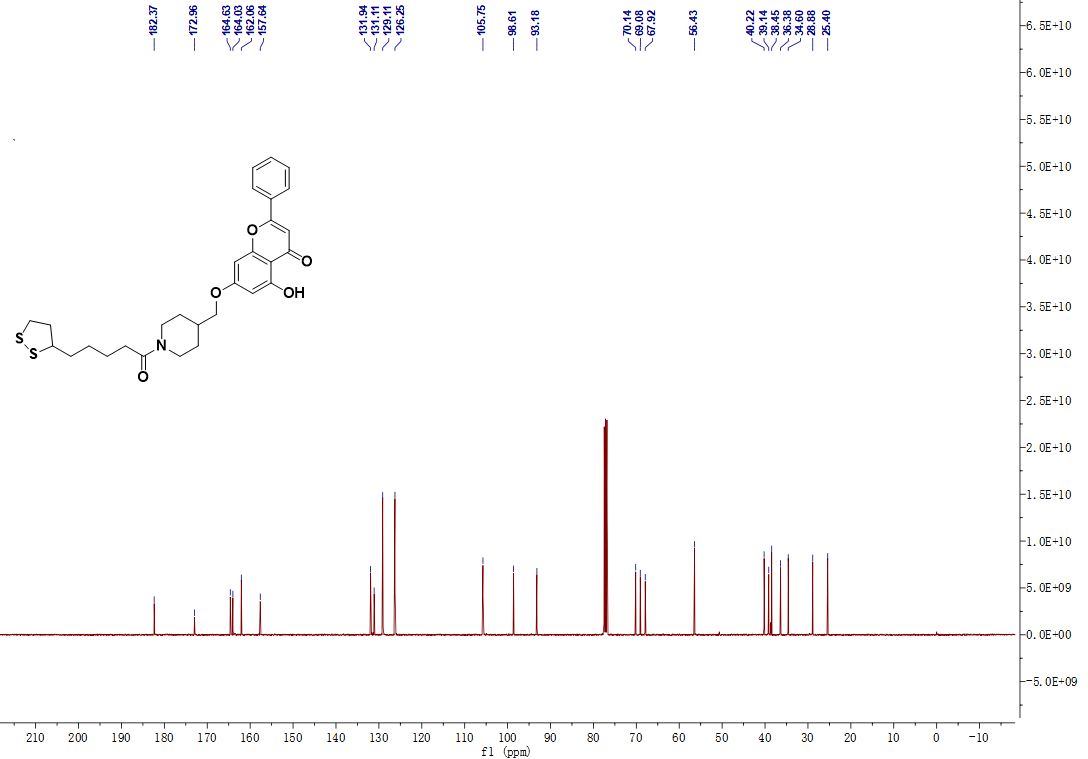
**

^13^C NMR Spectrum of Compound **4c**

**
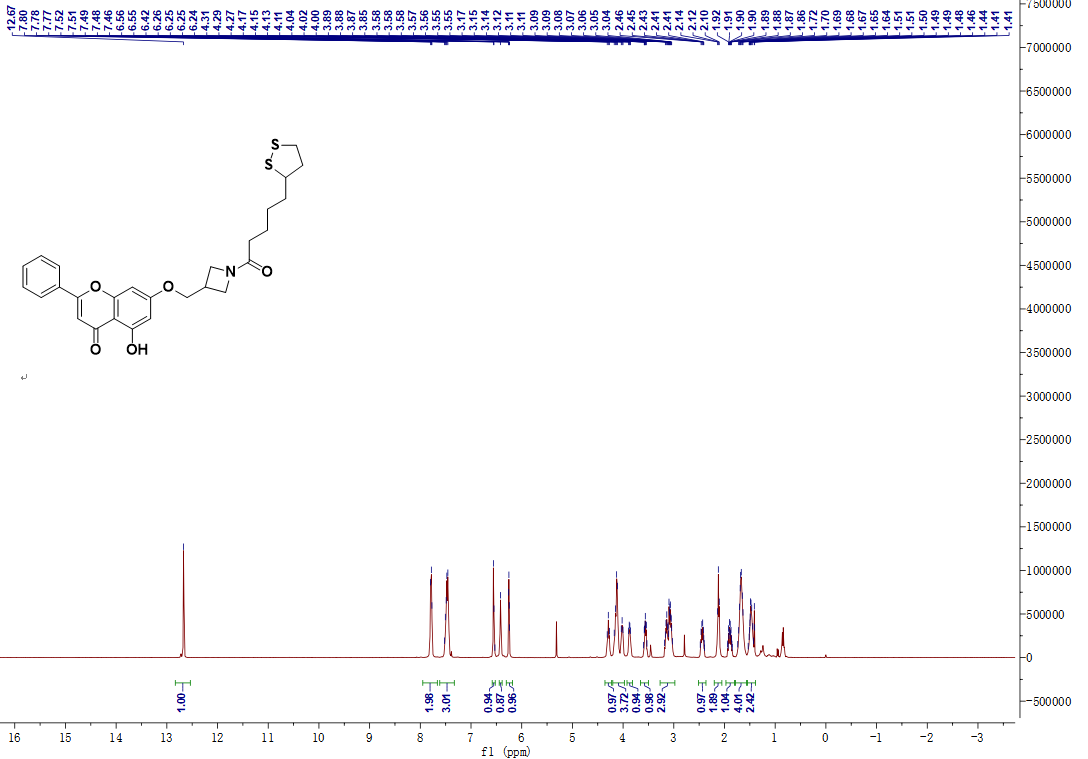
**

^1^H NMR Spectrum of Compound **4d**

**
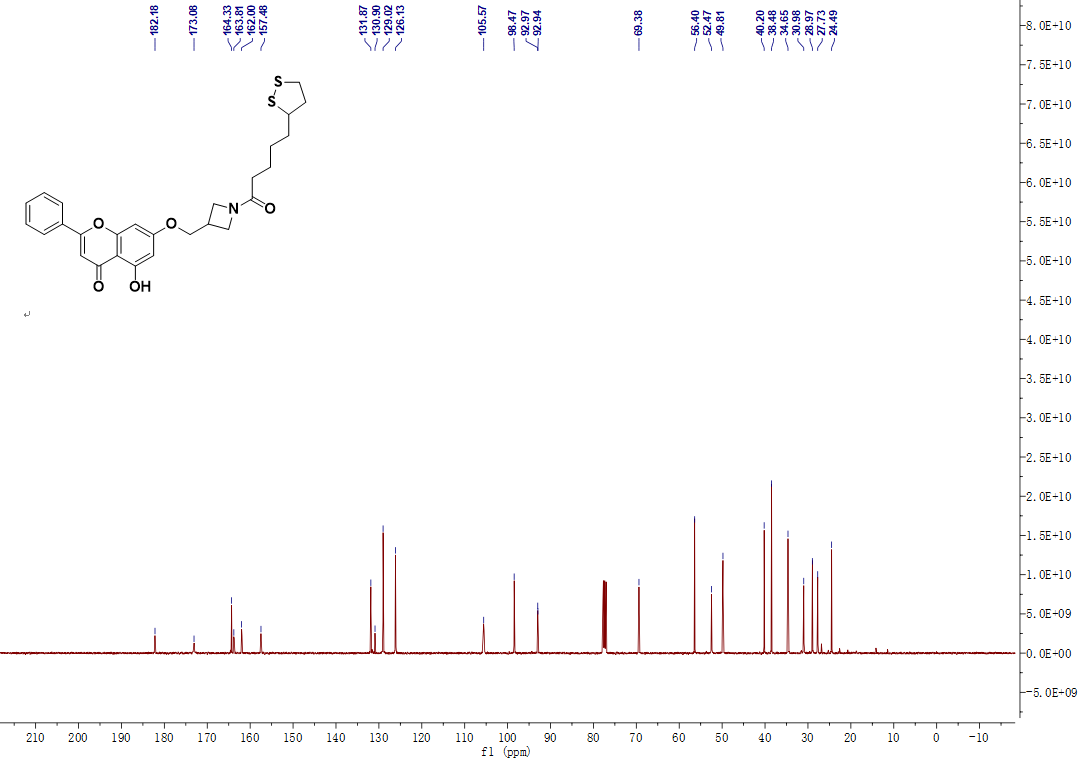
**

^13^C NMR Spectrum of Compound **4d**


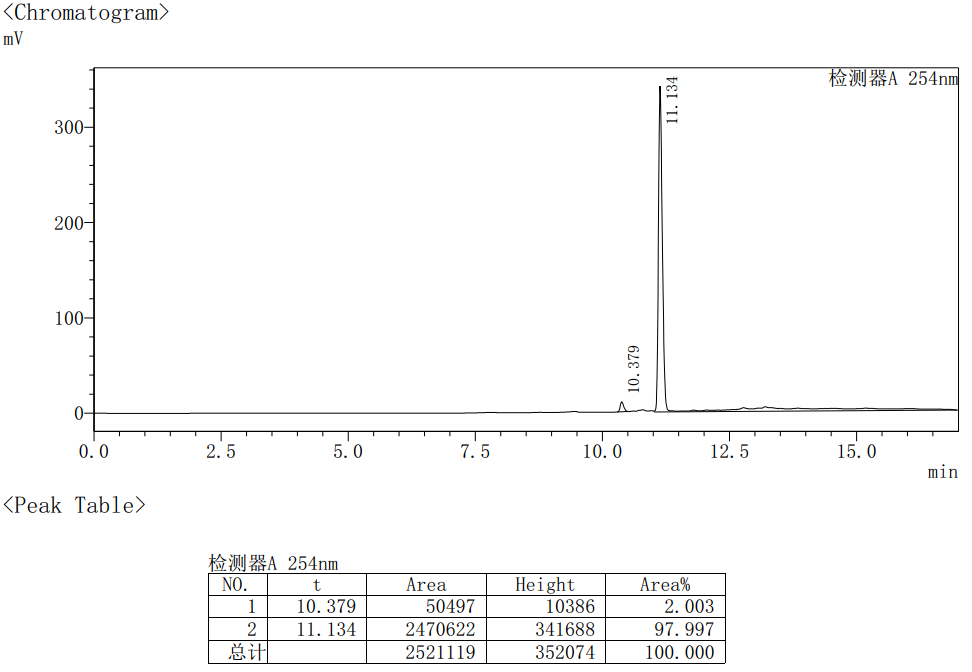


High-performance liquid chromatogram of Compound **4a**


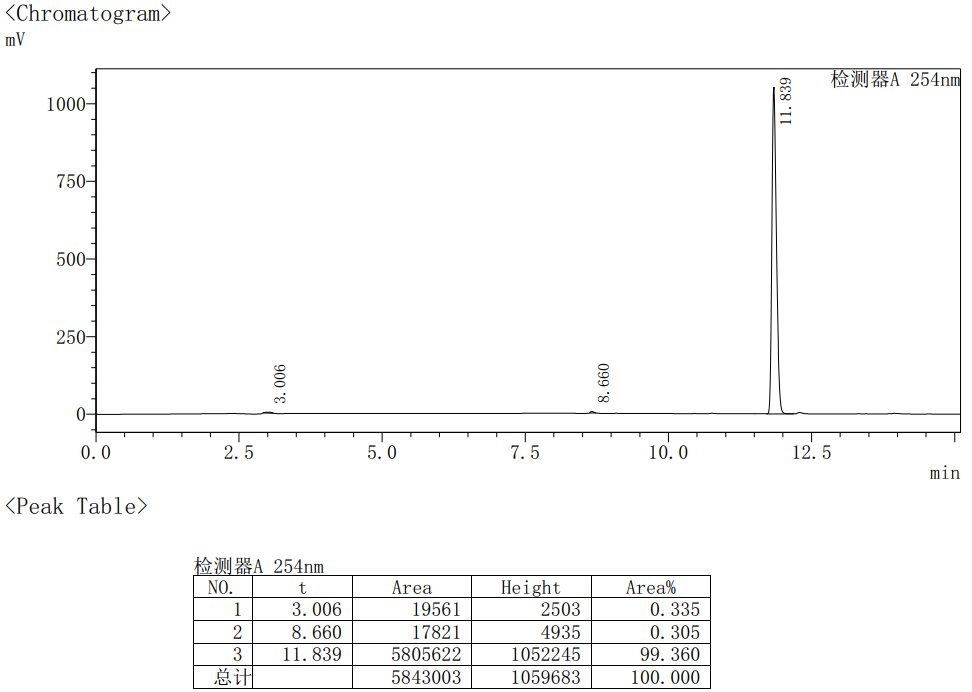


High-performance liquid chromatogram of Compound **4b**


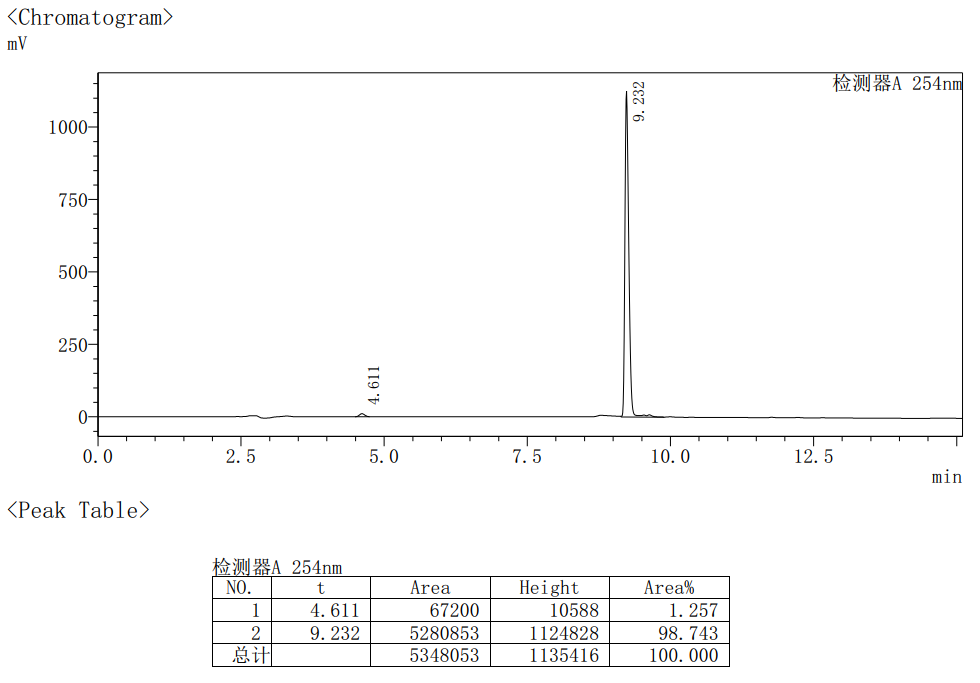


High-performance liquid chromatogram of Compound **4c**


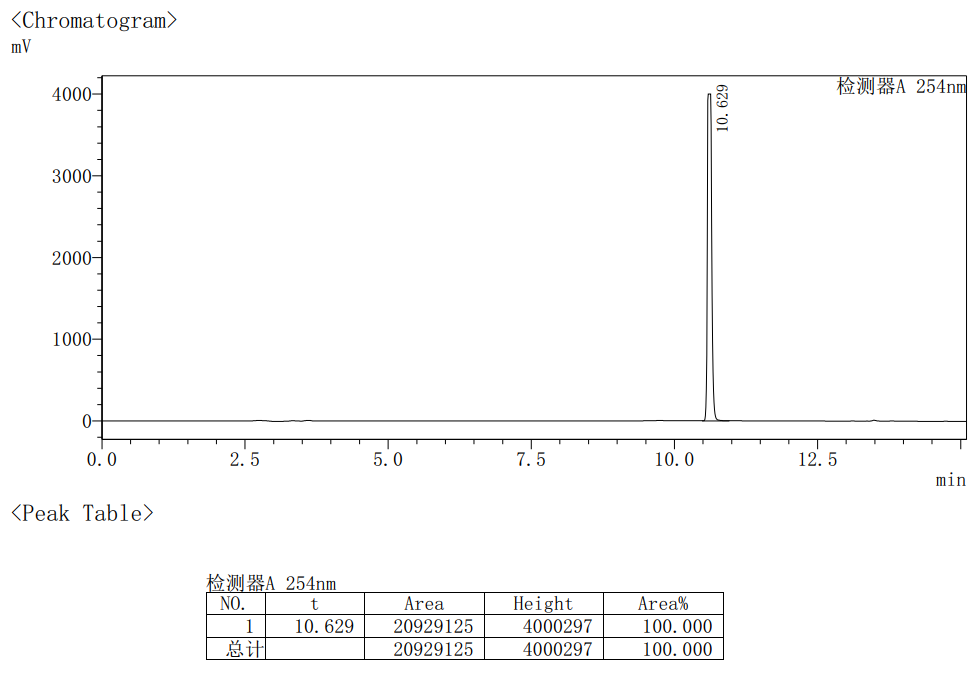


High-performance liquid chromatogram of Compound **4d**


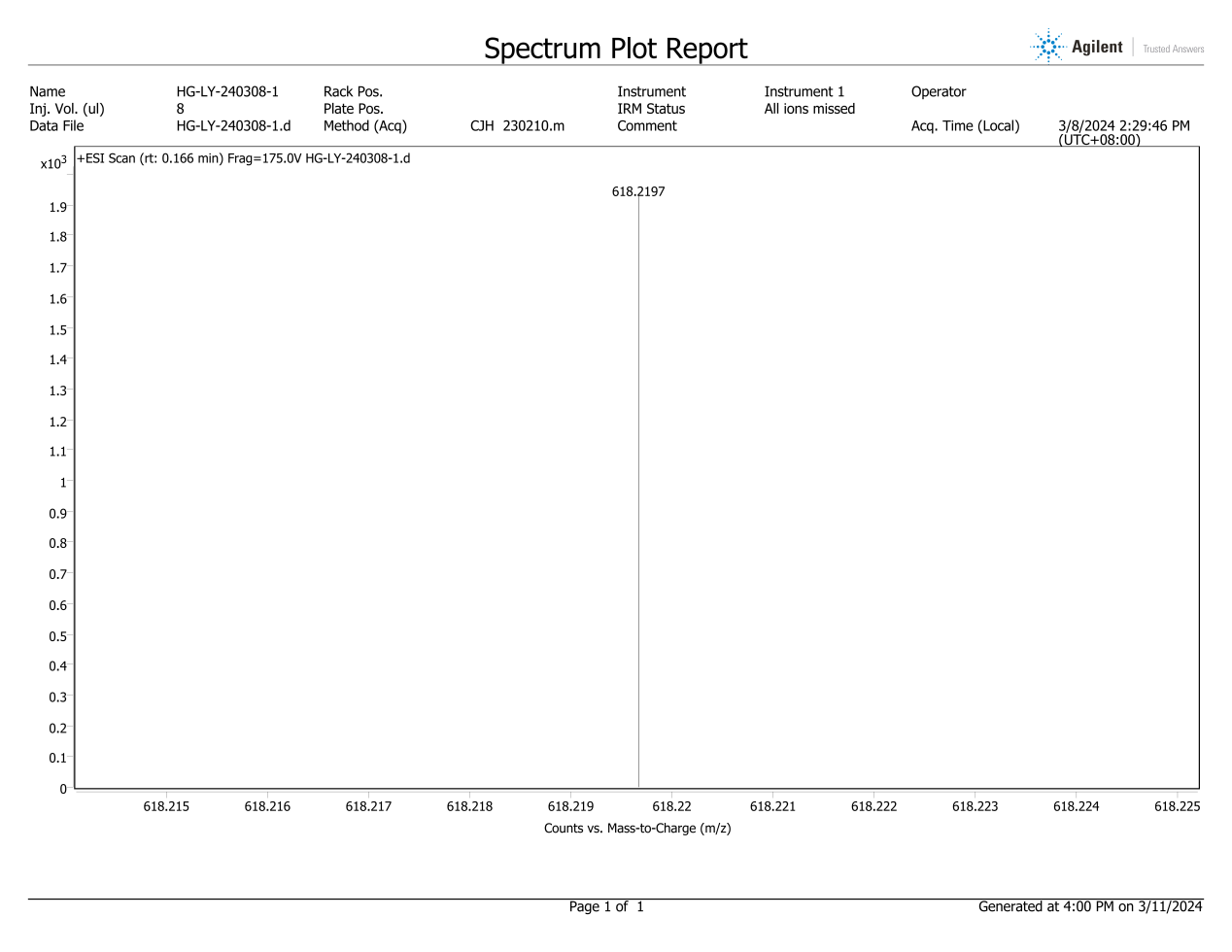


HR-MS (ESI) Spectrum of Compound **4a**


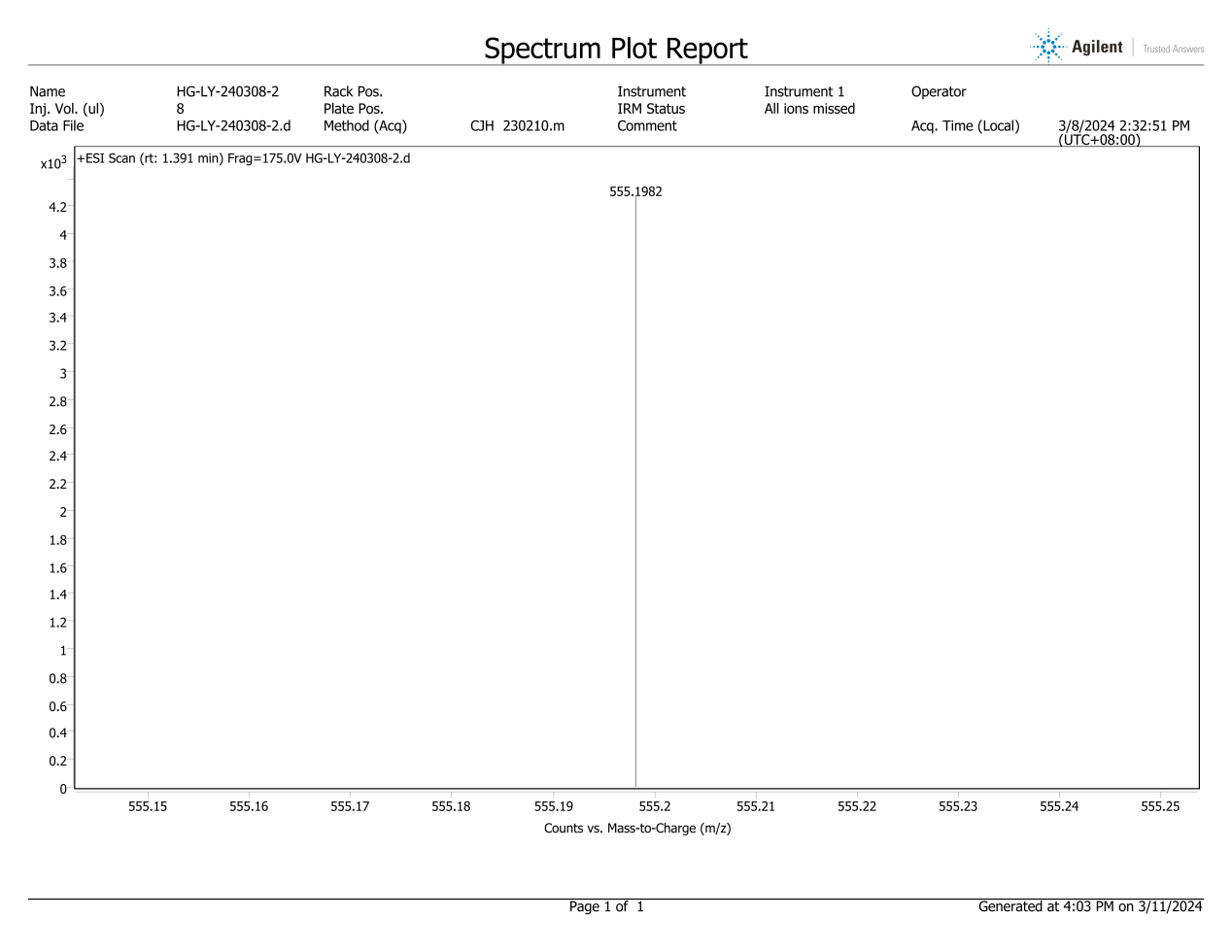


HR-MS (ESI) Spectrum of Compound **4b**

HR-MS (ESI) Spectrum of Compound **4c**


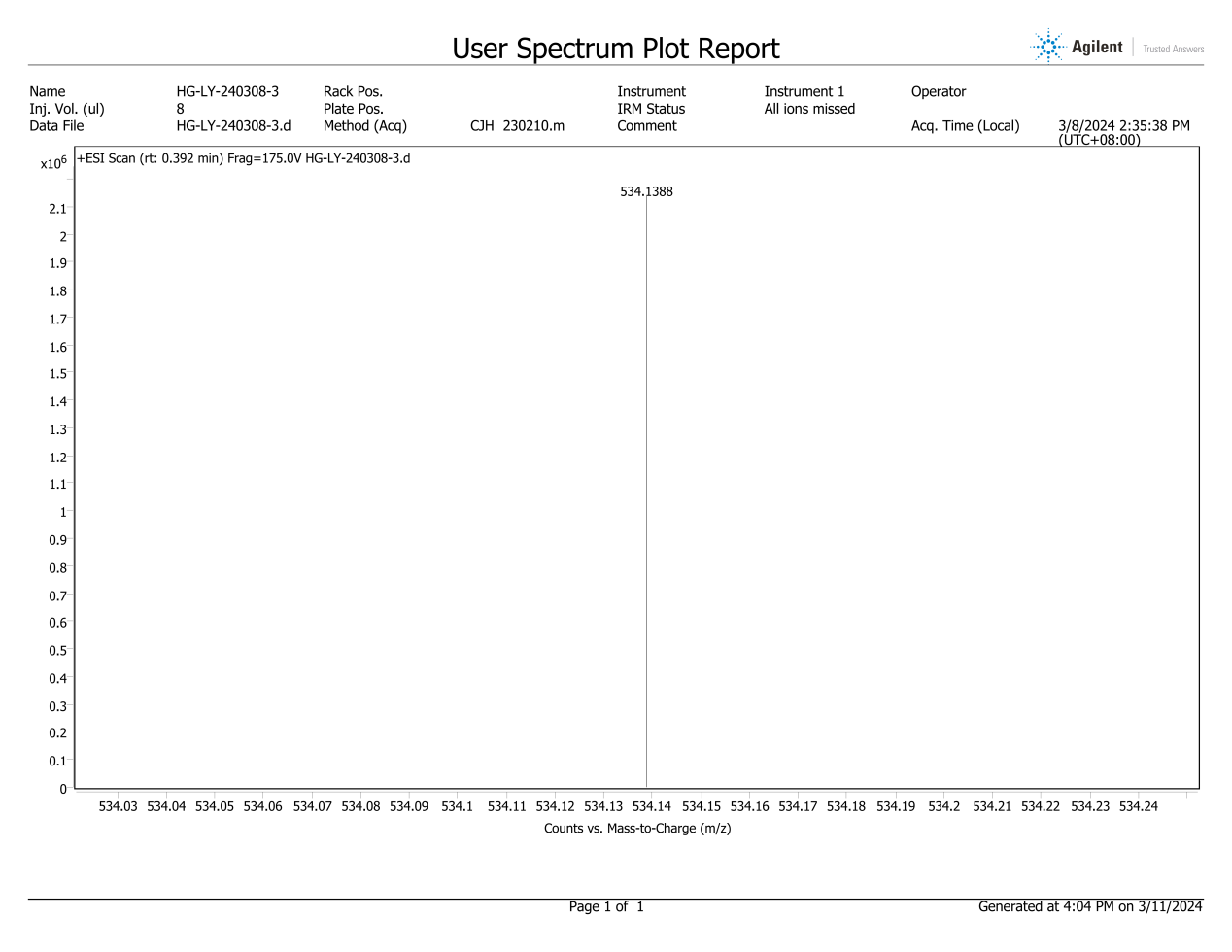


HR-MS (ESI) Spectrum of Compound **4d**

**Table S1** ADMET analysis of the synthesized compounds and chrysin

| Parameters / Samples | **4a** | **4b** | **4c** | **4d** | Chrysin |
| --- | --- | --- | --- | --- | --- |
| Physicochemical properties | | | | | |
| MW | 617.21 | 554.19 | 539.18 | 511.15 | 254.06 |
| nHA | 9 | 7 | 6 | 6 | 4 |
| nHD | 2 | 1 | 1 | 1 | 2 |
| nRot | 20 | 11 | 10 | 10 | 1 |
| nRing | 4 | 5 | 5 | 5 | 3 |
| MaxRing | 10 | 10 | 10 | 10 | 10 |
| nHet | 11 | 9 | 8 | 8 | 4 |
| fChar | 0 | 0 | 0 | 0 | 0 |
| nRig | 24 | 30 | 30 | 28 | 18 |
| Flexibility | 0.833 | 0.367 | 0.333 | 0.357 | 0.056 |
| Stereo Centers | 1 | 1 | 1 | 1 | 0 |
| TPSA | 116.460 | 83.220 | 79.980 | 79.980 | 70.670 |
| LogS | -4.637 | -5.051 | -5.136 | -4.668 | -3.506 |
| LogP | 4.519 | 5.114 | 5.955 | 5.492 | 3.580 |
| LogD | 3.336 | 3.739 | 3.834 | 3.662 | 2.947 |
| Absorption | | | | | |
| Caco-2 Permeability | -4.877 | -4.973 | -4.965 | -4.898 | -4.874 |
| MDCK Permeability | 3.3e-05 | 6.2e-0.5 | 2.9e-05 | 2.3e-05 | 1.3e-05 |
| Pgp-inhibitor | +++ | + | +++ | +++ | --- |
| Pgp-substrate | --- | ++ | + | --- | ++ |
| Human intestinal absorption | --- | -- | --- | --- | --- |
| 20% bioavailability | +++ | +++ | +++ | +++ | +++ |
| Distribution | | | | | |
| Plasma protein binding | 98.874% | 95.883% | 99.272% | 99.648% | 98.028% |
| Volume distribution | 0.793 | 2.247 | 0.875 | 0.478 | 0.493 |
| Blood-brain barrier penetration | --- | **-** | - | - | --- |
| The fraction unbound in plasms | 2.023% | 3.033% | 0.714% | 0.745% | 2.776% |
| Metabolism | | | | | |
| CYP1A2 inhibitor | + | + | + | ++ | +++ |
| CYP1A2 substrate | --- | +++ | + | - | -- |
| CYP2C19 inhibitor | +++ | ++ | ++ | +++ | ++ |
| CYP2C19 substrate | --- | **-** | --- | --- | --- |
| CYP2C9 inhibitor | +++ | ++ | ++ | ++ | ++ |
| CYP2C9 substrate | - | + | ++ | ++ | +++ |
| CYP2D6 inhibitor | ++ | +++ | ++ | ++ | ++ |
| CYP2D6 substrate | -- | +++ | ++ | ++ | + |
| CYP3A4 inhibitor | ++ | ++ | ++ | ++ | + |
| CYP3A4 substrate | -- | + | -- | -- | -- |
| Excretion | | | | | |
| Clearance | 10.892 | 9.508 | 8.548 | 9.196 | 5.131 |
| T_1/2_ | 0.434 | 0.050 | 0.058 | 0.067 | 0.787 |
| Toxicity | | | | | |
| hERG blockers | + | ++ | - | -- | --- |
| Human hepatotoxicity | -- | + | ++ | + | --- |
| Drug-induced liver injury | ++ | ++ | + | + | ++ |
| Skin sensitization | +++ | +++ | +++ | +++ | +++ |
| Rat oral acute toxicity | --- | --- | -- | -- | --- |
| Carcinogencity | + | -- | - | + | - |

**Abbreviation:**

nHA: Number of hydrogen bond acceptors; nHD: Number of hydrogen bond donors; nRot: Number of rotatable bonds; nRing: Number of rings; MaxRing: Number of atoms in the biggest ring; nHet: Number of heteroatoms; fChar: Formal charge; nRig: Number of rigid bonds; Flexibility: nRot/nRig; TPSA: Topological polar surface area; LogS: Log of the aqueous solubility; LogP: Log of the octanol/water partition coefficient; LogD: LogP at physiological pH 7.4.

**Tips:**

For the classification endpoints , the prediction probability values are transformed into six symbols: 0-0.1(---), 0.1-0.3(--), 0.3-0.5(-), 0.5-0.7(+), 0.7-0.9(++), and 0.9-1.0(+++).
